# Precision Medicine to Treat Advanced Gastroesophageal Adenocarcinoma: A Work in Progress

**DOI:** 10.3390/jcm9093049

**Published:** 2020-09-22

**Authors:** Valentina Gambardella, Tania Fleitas, Noelia Tarazona, Federica Papaccio, Marisol Huerta, Susana Roselló, Francisco Gimeno-Valiente, Desamparados Roda, Andrés Cervantes

**Affiliations:** 1 Department of Medical Oncology, Biomedical Research Institute INCLIVA, University of Valencia, Blasco Ibañez 17, 46010 Valencia, Spain; vgambardella@incliva.es (V.G.); tfleitas@incliva.es (T.F.); noetalla@incliva.es (N.T.); fede.papaccio@yahoo.it (F.P.); mhuerta@incliva.es (M.H.); srosello@incliva.es (S.R.); fgimenovaliente@gmail.com (F.G.-V.); droda@incliva.es (D.R.); 2 Instituto de Salud Carlos III, CIBERONC, 28000 Madrid, Spain

**Keywords:** advanced gastric cancer, precision medicine, new drug development

## Abstract

Gastroesophageal adenocarcinoma (GEA) represents a heterogeneous disease and, when diagnosed as locally advanced or metastatic, it is characterized by poor prognosis. During the last few years, several molecular classifications have been proposed to try to personalize treatment for those patients diagnosed with advanced disease. Nevertheless, despite the great effort, precision medicine is still far from being a reality. The improvement in the molecular analysis due to the application of high throughput technologies based on DNA and RNA sequencing has opened a novel scenario leading to the personalization of treatment. The possibility to target epidermal growth factor receptor (HER)2, Claudine, Fibroblast Growth Factor Receptors (FGFR), and other alterations with a molecular matched therapy could significantly improve clinical outcomes over advanced gastric cancer patients. On the other hand, the development of immunotherapy could also represent a promising strategy in a selected population. In this review, we sought to describe the novel pathways implicated in GEA progression and the results of the molecular matched therapies.

## 1. Introduction

Gastroesophageal adenocarcinoma (GEA) represents the fifth most frequent tumor and the third leading cause of cancer-related deaths worldwide. In 2018 GEA was diagnosed in about 1,000,000 people, causing death in 783,000 patients [1]. GEA is characterized by a worldwide variation in incidence, with a peak in Japan and South Korea, and lower incidence observed in the United States, Canada, India, and Middle Eastern countries. Beyond their higher rates of gastric cancer, Asian patients tend to have more distal gastric tumors, often associated with H. pylori, with less frequent gastroesophageal junction (GEJ) tumors, adenocarcinomas of the esophagus or Barrett’s esophagus disease [2]. This geographical distribution is a reflection of multifactorial etiology related mainly to genetic susceptibility, diverse strains of H. pylori, dietary factors, and principally differences in the tumor–immune microenvironment [3,4]. As symptoms during early cancer development are generally mild and unspecific, late diagnosis is frequent, contributes to the high mortality observed in countries without screening programs [5,6]. GEA is recognized as a highly heterogeneous disease. Despite concerted efforts to develop comprehensive molecular classifications for GEA aimed at offering a precision approach to patients, only a few drugs have been approved. The aim of this article is to review the state of the art in precision medicine for advanced GEA.

## 2. From Morphological to Molecular Classifications

Historically, Lauren classified gastric cancers into intestinal, diffuse, and indeterminate/mixed histology [7]. The most common type is intestinal, which tends to form glandular structures and is often associated with intestinal metaplasia and H. pylori infection. Conversely, diffuse-type tumors are characterized by non-cohesive scattered cells, sometimes associated with the presence of signet-ring cells. These tumors trends to be locally aggressive with a higher rate of peritoneum invasion and a lower response rate in neoadjuvant studies, when treated with platinum-based therapies without taxanes [8,9]. The World Health Organization (WHO) further classified gastric cancer into tubular, papillary, mucinous, poorly cohesive (including Lauren diffuse type), and mixed variants. Nevertheless, neither stratification has helped characterize patient outcomes or guide treatment approach.

Further research focus has been directed at analyzing predictive biomarkers and targetable drivers. Molecular studies have been led by The Cancer Genome Atlas (TCGA) and also by the Asian Cancer Research Group (ACRG) [10,11]. TCGA studied both adenocarcinoma and squamous cell carcinoma spanning the stomach and esophagus while the ACRG focused on gastric adenocarcinoma across the Asian population. TCGA proposed a comprehensive molecular classification of GEA according to genomic, transcriptomic and proteomic data into four different subgroups, respectively, named as chromosomally instability (CIN), genomically stable (GS), Epstein–Barr Virus (EBV) and microsatellite instability (MSI). More than 50% of tumors belong to the CIN subgroup, which is mainly characterized by receptor tyrosine kinase (RTK) alterations [10]. Despite this, apart from epidermal growth factor receptor (HER)2 amplification, MSI and EBV, no other molecular alterations have been used in the clinic as effective predictors for treatment decision-making (Table 1).

The ACRG [11] group developed a different classification, considering also four groups based on array-based gene-expression profiling: MSI (22.7%), microsatellite stable with epithelial-to-mesenchymal transition (MSS/EMT; 15.3%), MSS with TP53 mutations (26.3%), and MSS without TP53 mutations (35.7%). The MSS/EMT class was enriched in diffuse-type tumors classified as GS by TCGA, and MSS/TP53+ was enriched for EBV+ tumors. ACRG analysis found inferior survival among the MSS/EMT+ group. Although no survival associations were seen in the TCGA study, potentially owing to the therapeutic heterogeneity of the internationally diverse sample set, subsequent studies applying the TCGA classes to two large independent cohorts demonstrated that EBV+ tumors have superior survival and GS tumors have inferior survival [12].

Tumor heterogeneity is the main obstacle to progress in precision medicine for GEA patients. Comprehensive molecular assessment sequencing primary and metastatic GEA has revealed marked differences in genomic aberrations, with frequent discrepancies between findings in primary versus metastatic tumors. When gene mutations were specifically analyzed, 20% could be found only in the primary tumor, while a similar proportion was found only in metastatic tumors. The rest (60%) of mutations detected were shared by both primary and metastatic. Turning to gene amplifications, 32% are seen only in primary tumors, 31% in metastatic tumors and only 37% were shared by both. This observed discrepancy in genomic aberrations led to treatment modification in almost a third of patients [13,14]. Liquid biopsies assessing cell-free DNA have the potential to help optimize therapy selection.

## 3. Targeting HER2 beyond Trastuzumab: Novel Steps towards Precision Medicine

HER2 receptor is a member of the epidermal growth factor receptor (HER) family consisting of four members (HER1 (ErbB1, EGFR), HER2 (ErbB2), HER3 (ErbB3) and HER4 (ErbB4)), activated by spontaneous homo/heterodimerization [15]. HER2 is amplified in several tumors, such as breast, colorectal and GEA [16], where it accounts for about 7–34% of all cases. HER2 overexpression is more highly detected among tumors of the GE junction (GEJ) and varies across different histological subtypes and differentiation grades [17]. HER2-amplified tumors are mostly well- or moderately-differentiated intestinal subtypes [18]. Despite identification of HER2 amplification in GEA, the use of HER2 blocking agents has not achieved the outstanding results shown in breast cancer (Table 2.). Trastuzumab, a monoclonal antibody inhibiting HER2 activation by binding to the extracellular domain IV of HER2, improves clinical outcomes over HER2-amplified localized and advanced breast cancer, both as a single agent and in combination with chemotherapy.

A randomized multicenter trial (ToGA) assessed the addition of trastuzumab to platinum-based chemotherapy in HER2-amplified patients with locally advanced or metastatic GEA [19]. Patients were randomized to receive trastuzumab plus platinum-based chemotherapy (capecitabine or 5-fluorouracil plus cisplatin) versus chemotherapy alone. In the experimental arm, patients experienced a substantial improvement in all outcomes and particularly in overall survival (OS). Median OS was increased by 2.7 months in the trastuzumab-containing arm (13.8 versus 11.1 months, Hazard ratio (HR) 0.74, P.0.0046) [19]. Based on these results, the combination of trastuzumab plus platinum-based chemotherapy represents the gold standard for advanced HER2-amplified GEA. The extent of improvement was related to the degree of amplification, as demonstrated in an exploratory post-hoc analysis. The subset of tumors with HER2 immunohistochemistry (IHC) 3 or IHC2/FISH+ reached a median OS of 16 months in the trastuzumab group versus only 11.8 months in the chemotherapy control arm (HR 0.68, 95% CI 0.5–0.83). These results suggest that trastuzumab provides the best therapeutic benefit in strongly HER2-amplified patients [20].

These findings highlight the need for adequate patient selection. HER2 amplification is highly heterogeneous across patients and even intratumorally [21], a phenomenon that could account for the general lack of benefit when anti-HER2 drugs are administered without correct scoring by a dedicated pathologist. The level of HER2 amplification in metastatic GEA by FISH has been shown to predict response to trastuzumab [22]. Beyond IHC and FISH, when HER2 amplification was determined by next-generation sequencing (NGS) in tumor samples or in plasma, analyzing cell-free circulating tumor DNA, response to antiHER2 agents directly correlated with level of expression observed in plasma samples.

Other HER2 blocking drugs were successively tested to evaluate their potential in HER2-amplified and advanced GEA patients (Table 2). Disappointingly, no benefit in overall survival was achieved in patients diagnosed with locally advanced or metastatic GEA when treated with lapatinib, either as a single agent or in combination with platinum-based chemotherapy (CT) pertuzumab or TDM-1. Lapatinib, a dual EGFR/HER2 small tyrosine kinase inhibitor, was tested as first-line treatment in a phase III, double-blinded study (LoGic Trial) and failed to demonstrate OS improvement [23]. Lapatinib also failed to improve OS in a second-line trial in Asian patients (TyTAN trial) despite a significant increase in response rate. [24]. Based on the good results achieved in advanced HER2-amplified breast cancer patients, we also tested pertuzumab, a humanized monoclonal HER2-targeted antibody that binds to a different epitope on the HER2 receptor than trastuzumab. In a phase III trial (JACOB), the combination of trastuzumab, pertuzumab and platinum-based (CT) was tested as a first-line treatment in HER2 amplified GEA patients [25] and failed to improve survival.

Trastuzumab emtansine (TDM-1), an antibody-drug conjugate, was also tested as second line versus paclitaxel in patients progressing after first-line trastuzumab-containing treatment, showing no benefit in OS in the phase II/III GATSBY trial [26]. Similarly, when trastuzumab was tested in combination with paclitaxel beyond tumor progression in a phase II trial enrolling patients who experienced progression on first line with trastuzumab and platinum-based CT, no benefit was found. In this phase II randomized trial, trastuzumab beyond progression, a common approach used in HER2 amplified BC did not improve progression free-survival (PFS) in advanced HER amplified GEA patients [27]. One possible cause of treatment failure could have been the significant loss of HER2 amplification after trastuzumab blockade, already described in HER2 amplified BC [22,28]. Several other mechanisms of resistance have been addressed to explain the lack of benefit of other antiHER2 agents. Among them, the most significant could be intratumor heterogeneity, as well as the presence of emerging molecular alterations, such as PI3K, MAPK activation and MET or FGFR aberrations [13,17,29,30,31].

Interestingly, an elegant translational study found that oral pan-HER inhibitor afatinib could rescue patients progressing after trastuzumab when they co-expressed HER2, EGFR or MET amplifications, paving the way for multi-targeted agents according to personalized profiles [13].

Among other antiHER2 agents under development, margetuximab, an Fc-modified chimeric monoclonal antibody, has shown promising results in early-phase clinical studies for HER2-expressing solid tumors, including low HER2-expressing gastric cancer [32]. In a phase 1b–2 trial in locally advanced HER2-amplified, PD-L1 unselected GEJ, patients who experienced disease progression to trastuzumab and at least two previous lines of CT were enrolled to receive margetuximab and pembrolizumab. This combination showed acceptable safety and tolerability and objective responses were observed in 18.4% of evaluable patients, suggesting synergistic anti-tumor activity with checkpoint inhibitors [33]. Preclinical and clinical evidence support combining pembrolizumab with trastuzumab and cytotoxic chemotherapy to treat HER2-positive cancers [34]. In several analyses performed in tumor samples of GEA patients treated with trastuzumab, it was possible to observe upregulation of PD-1 and enhanced gene expression signatures of immune infiltration, [35] which could suggest a potential benefit deriving from checkpoint inhibitors. Moreover, the combination of trastuzumab with pembrolizumab could increase T-cell response [36,37]. On the strength of these findings, this combination has a promising biological rationale.

In a phase II trial, patients diagnosed with locally advanced or metastatic HER2-amplified GEA were treated with pembrolizumab, trastuzumab, fluoropyrimidine and platinum as first-line therapy. Twenty-six (70%) of 37 patients were progression-free at 6 months and 17% achieved a complete response. The 91% response rate and median overall survival of 27.3 months were also higher than previously reported for chemotherapy plus trastuzumab, the existing first-line standard [19]. Treatment-related adverse events were similar as with combinations of trastuzumab with chemotherapy and pembrolizumab with chemotherapy [38,39]. PD-L1 combined positive score was not predictive of outcome in this study population; nevertheless, patients with HER2 amplification had more durable responses to trastuzumab-based combination therapy than HER2-positive patients who did not have HER2 gene amplification by next-generation sequencing. A phase III trial of pembrolizumab versus placebo in combination with trastuzumab and chemotherapy is ongoing.

Interesting results have recently been achieved with another antibody-drug conjugate. Trastuzumab deruxtecan (DS-8201) consists of a humanized, monoclonal, anti-HER2 antibody binding a cytotoxic topoisomerase I inhibitor by means of a cleavable, tetrapeptide-based linker [40]. This drug is stable in plasma [41], but is highly cleaved in cancer cells [42]. The most appealing characteristic of this conjugate is that it binds even to cells with lower levels of HER2 expression [43] due to the ability of the released payload to diffuse across cell membranes. Probably owing to this dynamic feature, trastuzumab deruxtecan represents a good strategy for heterogeneous tumors, such as gastric cancer in which HER2 overexpression may vary from cell to cell or even across different metastatic locations within the same patient [19,44]. In a phase II trial design, after encouraging results obtained in a previous phase I, patients diagnosed with advanced GEA who had received at least two previous lines were stratified into high and low HER2 and were randomized 2:1 to receive trastuzumab deruxtecan versus the treating physician’s choice of irinotecan or paclitaxel [45,46]. Objective response was significantly higher in the experimental group than with chemotherapy (51% versus 14%). Ten patients (8%) in the experimental arm had a confirmed complete response versus none in the chemotherapy arm. Patients on trastuzumab deruxtecan gained significantly in overall survival (median OS, 12.5 months versus 8.4 months. HR = 0.59; *p* = 0.01). Greater efficacy of trastuzumab deruxtecan over chemotherapy was confirmed in patients with the highest level of HER2 expression (Response Rate 58% versus 29%) [46]. However, in tumors with lower HER2 expression levels, a lower response rate was determined. The more efficient linker-payload system of trastuzumab deruxtecan, ten-fold more potent than SN-38, may contribute to the differing treatment outcomes versus TDM-1 [47,48]. Interstitial lung disease has been observed in 10% of patients receiving trastuzumab deruxtecan, most classified as grade II, although some were severe. Monitoring this trend and understanding ways of preventing and treating it is vital before recommending this drug-conjugate antibody for general use.

## 4. Targeting MET, EGFR, PI3K/AKT/mTOR Pathways and NTRK Fusions

Phase III trials with other targeted therapies based on molecular features other than HER2 have achieved disappointing results in GEA (Table 3) [2,49]. The epidermal growth factor receptor (EGFR) is amplified in about 5% and the receptor is overexpressed in between 30–50% of cases [50]. Both cetuximab and panitumumab, approved anti-EGFR monoclonal antibodies in advanced colon cancer, have been tested. However, in two randomized phases III trials, no improvement in clinical outcomes was detected when the anti-EGFR treatment was added to a first line of a platinum-based chemotherapy [51,52]. These anti-EGFR antibodies might have decreased the tolerance of the backbone CT in first line, leading to dose delays and reduction which could have had a detrimental effect. Beyond first line, the anti-EGFR tyrosine kinase inhibitor gefitinib showed no clinical benefit versus placebo in tests [53]. As a general limitation, no molecular patient selection was properly performed. The subset analysis of both COG [53] and EXPAND trials [51] underlined a potential effect in EGFR-amplified patients. Nevertheless, these results should be confirmed prospectively.

The results of MET inhibition have also been discouraging. MET amplification is detectable in about 6% and overexpressed in 25–60% of GEA [10,49]. When monoclonal antibodies such as onartuzumab [54] and rilotumumab [55] were added to first-line chemotherapy, no clinical benefit was observed. Moreover, similarly negative results were obtained when tyrosine kinase inhibitors such as AMG 337 were administered in a heavily treated population of MET-amplified patients. Another molecular alteration widely present across GEA patients is activation of the PI3K–AKT–mTOR pathway. Everolimus, a mTOR inhibitor approved in breast cancer, was tested on unselected patients, showing no clinical benefit in OS beyond the first line [56].

Given the evidence of the complex molecular landscape of GEA, an umbrella platform [57] has been designed with preplanned genomic biomarker analyses to assign advanced GEA patients to molecularly matched therapies. Several biomarker groups were identified based on RAS alterations, TP53, PIK3CA mutations, MET and PIK3CA amplification, etc. Of the whole screened population, only 14.7% received biomarker-assigned drug treatment. The highest response rate was observed in patients with MET amplification treated with savolitinib, a potent small-molecule reversible MET kinase inhibitor. This strategy obtained encouraging response rates and survival when compared with conventional second-line standard chemotherapy, especially in patients presenting high MET expression enriched for higher MET copy number. Circulating tumor (ctDNA) analysis demonstrated good correlation between high MET copy number by ctDNA and response to MET inhibitors. Further results are awaited.

As NTRK has been recently identified as a relevant molecular driver over solid tumors, despite the low incidence over GEA, the molecular evaluation searching for NTRK fusion, could be an option in patients with a good performance status [58].

## 5. The Role of Stemness and Metalloprotease in GEA

As interesting preclinical data suggested the potential role of stemness pathways in GEA development and progression, the inhibition of STAT3, a principal actor of this pathway, was also tested. Nevertheless, when NAPA, a STAT3 inhibitor, was added to Paclitaxel, no clinical benefit was demonstrated in a phase III trial enrolling pre-treated GEA patient [58]. Disappointing results were also observed in a phase III trial randomizing patients to receive andecaliximab, a monoclonal antibody targeting matrix metalloproteinase 9 (MMS9), combined with CT in pretreated GEA patients. Despite the potential role of MMS9 in GEA development and progression, the inhibition of this target was no longer able to increase PFS or OS [59]. The lack of predictive biomarker could have negatively influenced the potential role of these drugs.

## 6. Angiogenesis-Targeting Drugs

Although antiangiogenic therapies are the current standard of care in other gastrointestinal tumors, addition of these agents did not show a benefit in unselected patients with GEA. In first line, bevacizumab improved overall response rate and PFS, but not OS, when added to chemotherapy for the first-line treatment of advanced gastric cancer [60]. In the same setting, ramucirumab was not able to improve clinical outcomes, yet demonstrated significant albeit modest OS benefit in the second line, both as monotherapy and combined with docetaxel [61,62,63]. In an Asian population, apatinib, a tyrosine kinase inhibitor of VEGFR2, showed improvement in PFS and OS when used in patients who experienced disease progression to two or more lines [64]. No biomarkers able to predict response to antiangiogenics have been so far identified.

## 7. The Search for Biomarkers for Precision Immunotherapy

Immunotherapy has revolutionized the treatment of many solid tumors; nonetheless, no biomarker for patient selection has been clearly identified as yet [65,66]. Among GEA patients, the PD-L1 combined positivity score (CPS), consisting of immunohistochemistry-detected protein in at least 1% of cells in the tumor or surrounding stroma, is often used for candidate selection in pembrolizumab-related trials. In the phase II KEYNOTE-059 trial, evaluating pembrolizumab as third-line treatment for metastatic gastric cancer, the overall response rate was 22.7% in PD-L1–positive tumors, with a median response duration of 8.1 months [38]. However, response rate was only 16.2% in PD-L1-negative tumors. On the other hand, results obtained testing another anti-PD-1 inhibitor, nivolumab, were similar to pembrolizumab, yet no relation with PD-L1 expression was found. In the phase III ATTRACTION-2 trial including Asian patients, nivolumab monotherapy led to an overall response rate ORR of 11% and significantly increased 12-month OS to 27% versus 11% with a placebo (HR: 0.63; *p* = 0.0001). This survival benefit was independent of PD-L1 positivity [65]. When nivolumab was tested in a western population as a single agent or added to ipilimumab, ORR was higher in PDL1-positive tumors (27%) than in PDL1-negative tumors (12%) [66].

MSI-H and EBV-positive tumors each comprise approximately 5% of metastatic and 20–30% of localized gastric cancers [10]. The MSI-H subtype is characterized by hypermutation, frequently on *KRAS, PI3K, PTEN, mTOR, ALK,* and *ARID1A*, resulting from silenced DNA mismatch repair proteins, such as MLH1. EBV-positive gastric cancer is characterized by high PD-L1 and 2 expression, *PIK3CA* and *CDKN2A* mutations, and *JAK2* amplification. Both MSI-H and EBV-positive types, regardless of histology, are considered the most immunogenic subtypes, susceptible to treatment with checkpoint inhibitors [67]. A recent phase II study testing pembrolizumab in metastatic gastric cancer patients described a notable, durable response in the MSI and EBV subtypes [68]. In MSI tumors, checkpoint inhibitor antitumor activity is associated with high tumor mutation load and neoantigen formation [68], with response rates for MSI gastric cancer ranging from 57% to 86% [67,68]. EBV subtype tumors are associated with tumor immune-cell infiltration and high expression of PD-L1, and in a subgroup analysis of a phase II study, all EBV-positive gastric cancer patients responded to pembrolizumab [67].

Furthermore, correlation between gene expression profile and response to checkpoint inhibitors was assessed in two different cohorts of advanced GEA patients treated with immunotherapy, used as a test and validation cohort. High alternate promoter utilization tumors exhibited decreased markers of T-cell anti-cancer activity, causing immune evasion and lower responses to checkpoint inhibitors (8% versus 42%, *p* = 0.03). This alternative promoter utilization was confirmed in multivariate analysis as an independent predictor of survival in patients receiving checkpoint inhibitors, indicating the potential role of alternate promoter utilization as a predictive biomarker for immunotherapy [69,70].

The GEA tumor microenvironment is directly involved in tumor development and progression [71] and, for this reason, the combinations of checkpoint inhibitors and antiangiogenetic drugs is being tested. In a small phase II trial, the use of the combination of pembrolizumab and levantinib as I or II line showed, across HER2 negative Asiatic GEA, an objective response recorded in 69% of patients with an acceptable toxicity suggesting a potential role of the combination also in the MSS population that should be further explored [72,73]. The combination of nivolumab and regorafenib is also under evaluation [74]. Further results are awaited.

## 8. Novel Potentially Druggable Pathways: Tight Junction Proteins, Fibroblast Growth Factor Pathway and DNA Damage Repair Response

### 8.1. Claudin 18.2

Tight junction protein Claudin (CLDN) 18.2 has recently been identified as a possible target for GEA patients [75]. Physiologically, CLDN18.2 is buried in the tight junction supramolecular complex. However, due to changes occurring in the malignant transformation, tight junctions expose CLDN18.2 epitopes on the surface of tumor cells [75], making it possible to target it. Zolbetuximab is a chimeric IgG1 monoclonal antibody that binds to CLDN18.2 on the surface of tumor cells inhibiting cell survival and proliferation through antibody-dependent cellular cytotoxicity (ADCC) and complement-dependent cytotoxicity (CDC) [76].

In a phase II trial, patients diagnosed with advanced GEA who had progressed to at least one previous chemotherapy line, whose tumors expressed CLDN18.2 (moderate–intense membrane staining intensity in >50% of cancer cells) were treated with zolbetuximab as monotherapy [77]. A total of 54 patients were enrolled. Zolbetuximab monotherapy was found to be well tolerated with only mild gastrointestinal adverse events and exhibited anti-tumor activity in patients with CLDN18.2- positive advanced gastric or GEJ adenocarcinomas, as already demonstrated when it was used as a single agent. Despite the small number included, among 43 evaluable patients, 4 achieved partial response (ORR 9%) and 6 (14%) had stable disease for a clinical benefit rate of 23%. In the subgroup of patients whose tumors expressed moderate or high CLDN18.2 level in >70% of tumor cells, ORR was 14%. Moreover, a randomized exploratory phase II trial (FAST) showed that zolbetuximab combined with platinum-based chemotherapy (EOX) confers a survival benefit over EOX alone in patients with CLDN18.2þ positive advanced gastric/GEJ cancer. These results warrant validation in a prospective randomized phase III design. Several phase III trials among Caucasian and Asian patients are currently underway to evaluate the role of zolbetuximab in improving OS in locally advanced and metastatic GEA in combination with a platinum-based chemotherapy.

### 8.2. FGFR Pathway

FGFR signaling is made up of four highly conserved transmembrane tyrosine kinases receptors (FGFR1–4) and FGFR5, which lack the intracellular kinase domain. All these receptors are activated by FGF, and participate in cell survival and proliferation [78,79]. Multiple trials studying diverse solid tumors have proposed the aberrant FGFR signaling pathway as a potential therapeutic target, and several inhibitors are under development (Table 4) [80,81,82,83,84,85,86,87,88,89]. The FGFR2 splice variant FGFR2b [89] is overexpressed in 2.5–31.1% of GEA [10]. In in vitro and in vivo models of GEA, FGFR2b overexpression has been related to both amplification and aberrant transcriptional upregulation of the FGFR2 gene [78], and in GEA, both FGFR2b overexpression and FGFR2 gene amplification have been associated with worse prognosis. FGFR2 gene amplification is associated with both chromosomal instability and genomically stable molecular subtypes [71].

In a phase II trial, patients diagnosed with advanced GEA presenting FGFR2 amplification or polysomy were randomized to receive AZD4547, an FGFR tyrosine kinase inhibitor, or paclitaxel. AZD4547 did not improve PFS over standard CT [88]. The lack of benefit could relate to the notorious intratumor heterogeneity for FGFR2 gene amplification and poor concordance between FGFR2 amplification/polysomy and FGFR2 expression [88]. Bemarituzumab (FPA144), is a humanized IgG1 monoclonal antibody that specifically inhibits cancer cells presenting the splice-variant FGFR2b binding of the ligands FGF7, FGF10, and FGF22 (25). In a phase I basket trial enrolling patients with advanced solid tumors and a specific cohort for GEA, it demonstrated single-agent activity as late-line therapy in patients with advanced-stage GEA, achieving partial response in 5 out of 28 patients. Bemarituzumab is currently being evaluated in combination with chemotherapy in a phase III trial as front-line therapy for patients with high FGFR2b-overexpressing advanced-stage GEA [89]. Apart from amplification, FGFR may exhibit other molecular alterations, and several novel drugs have been tested in solid tumors harboring mutations and gene rearrangements. The specific role of each one and their contribution in predicting drug response seems different for each specific molecular alteration presented in each of the four genes involved in the FGFR family [85]. Table 4 shows different FGFR inhibitors under development, describing their mechanisms of action as well as other characteristics including availability of predictive biomarkers.

### 8.3. DNA Damage Response Pathway in GEA

The identification of a molecular subgroup characterized by chromosomal instability (CIN) and aneuploidy underlines the possible role of DNA damage in GEA. In light of this, several trials have studied the use of PARP inhibitors. Despite promising phase II data [90], the phase III GOLD trial failed to show benefit from adding the PARP inhibitor olaparib to paclitaxel in the second-line setting [91]. These negative results across the overall unselected population might be explained by the lack of appropriate predictive biomarkers indicating PARP-dependency or homologous recombination defects (HRD). Unexpectedly, patients in whom immunohistochemistry detected loss of ATM expression causing potential sensitivity to PARP inhibitors, saw no benefit from olaparib either. Potential explanations include immature follow-up of the ATM-negative subgroup, and confounding of treatment by favorable prognostic factors enriched in ATM-low tumors, such as PD-L1 expression [91]. The data also underline the need for additional evaluation of HRD and replication stress as predictive biomarkers of response to PARP inhibitors. Platinum sensitivity may itself be a predictive biomarker, a concept that has led to a phase III trial of PARP inhibition versus placebo as maintenance therapy in locally advanced or metastatic gastric cancer that experienced response to a first line of platinum-based chemotherapy.

Maintenance strategies are also ongoing. In a phase III trial, advanced GEA patients, who have responded to first-line, platinum-based chemotherapy for at least 8 weeks, are randomized to placebo versus pamiparib, a novel PARP inhibitor. Other experimental agents, such as CHEK1, ATR, and WEE1 inhibitors are also under clinical testing. Among the CIN subgroup, some GEAs presented high amplification of KRAS or other alterations in cell-cycle regulators, and consequently several studies evaluating inhibition of RAS downstream effectors and CDK2 inhibitors are underway.

## 9. Conclusions

GEA presents as a very heterogeneous disease. The high number of molecular alterations, as reflected in the percentage of tumors belonging to the CIN subtype, makes a precision approach complicated. Nevertheless, the possibility of identifying certain drivers due to the implementation of omics in recent years has opened up novel opportunities in cancer patients. Among HER2-amplified tumors, more active molecules are leading to significantly improved benefits in these patients. The growing interest in tumor microenvironment, together with development of novel immunotherapies and combinations could also herald new approaches. The road towards a personalized approach is long, requiring further studies and breakthroughs in current knowledge.

## Figures and Tables

**Table 1:** Molecular features of GEA subgroups of TCGA analysis.

GS	EBV	MSI	CIN
*CDH1* and *RHOA* mutations	DNA hypermethylation	Hypermutation	RTK-RAS activation
*CLDN18-ARHGAP 26* fusion	*PIK3CA* and *ARID1A* mutations	*MLH1* silencing	*TP53* mutation
Cell adhesion	*JAK2* amplification	*RAS* activation	Cell cycle activation
	*CDKN2* silencing		
	Immune activation	Mitotic pathway	

GEA: Gastroesophageal adenocarcinoma; TCGA: The Cancer Genome Atlas.

**Table 2:** Randomized clinical trials testing anti-epidermal growth factor receptor (HER)2 blocking agents in HER2-amplified advanced GEA.

TRIAL.	Phase	Experimental Drug	Chemotherapy Backbone	Line of Therapy	Number of Included Patients	HR for OS	*p* Value	Response Rate	Increase in Median Survival
ToGA [19]	III	Trastuzumab	Cisplatin+5-FU/capecitabine	First	584	0.74	0.04	51% vs. 37% *p* = 0.0017	+2.8 months
LOGiC [23]	III	Lapatinib	Oxaliplatin/capecitabine +/−Lapatinib	First	545	0.91	0.35	53% vs. 39% *p* = 0.031	+1.7 months
TyTAN [24]	III	Lapatinib	Paclitaxel+/−Lapatinib	Second	261	0.84	0.20	27% vs. 9% *p* = 0.001	+2.1 months
GATSBY [26]	II/III	TDM-1	TDM-1 vs. Taxane	Second	345	1.15	0.85	NP	−0.7 months
JACOB [25]	III	Pertuzumab	Cisplatin+5-FU/ capecitabine /Trastuzumab +/− Pertuzumab	First	780	0.84	0.056	56% vs. 48% *p* = 0.026	3.3 months
DESTINY-Gastric01 [46]	II	Trastuzumab Deruxtecan	Trastuzumab Deruxtecan vs. Paclitaxel or Irinotecan	Third	187	0.59	0.01	51% vs. 14%	4.1 months

GEA: Gastroesophageal adenocarcinoma. HR: Hazard Ratio. OS: Overall survival. 5FU: 5-fluorouracile.

**Table 3:** Randomized trials targeting angiogenesis, EGFR and MET pathways in first line in GEA.

Trial	Chemotherapy	Experimental Drug	HR	Trial	Chemotherapy
AVAGAST [62]	Cisplatin+ capecitabine	Bevacizumab	0.87	0.10	+2.0 months
RAINFALL [63]	Cisplatin+5-FU/capecitabine	Ramucirumab	0.96	0.68	0.5 month
EXPAND [51]	Cisplatin+ capecitabine	Cetuximab	1.00	0.95	−1.3 months
REAL-3 [52]	Oxaliplatin+ epi- + capecitabine	Panitumumab	1.37	0.013	−2.5 months
RILOMET-1 [55]	Cisplatin+ epi+capecitabine	Rilotumumab	--	--	Stopped in futility analysis
METGASTRIC [54]	FOLFOX6	Onartuzumab	1.06	0.83	−0.6 months

GEA: Gastroesophageal adenocarcinoma. HR: Hazard Ratio, OS: Overall Survival, EGFR: Epidermal Growth Factor Receptor. 5FU: 5-Fluorouracile.

**Table 4:** FGFR inhibitors under development.

Compound	Type	Mechanisms of Action	Predictive Biomarkers	Clinical Phase of Development
Bemarituzumab	mAb	Inhibitor of FGF7, FGF10, and FGF22 ligand of the splice-variant FGFR2b		II, III
AZD4547	TKi	Potent and selective inhibitor of FGFR 1, 2, and 3		II
Infigratinib	TKi	Selective, ATP-competitive inhibitor of FGFR1, 2, and 3		I
E-7090	TKi	Oral and selective inhibitor of FGFR1, 2, and 3		I
LY2874455	TKi	Potent Pan FGFR inhibitor		I
Pemigatinib [81]	TKi	Potent inhibitor of FGFR1, 2, and 3	FGFR2 fusions	II
Rogaratinib [82]	TKi	Potent Pan FGFR inhibitor	FGFR1-3 mRNA expression	I
Futibatinib [83]	TKi	Potent and highly specific against wildtype FGFR1–4 as well as against some FGFR2 kinase domain mutations	FGFR2 fusions, FGFR1 mutations	I
Fisogatinib	TKi	Potent and selective inhibitor of FGFR4		I
H3B-6527	TKi	Selective and covalent inhibitor of FGFR4		I
Roblitinib	TKi	Potent and selective, reversible-covalent small-molecule inhibitor of FGFR4		I
Erdafitinib	TKi	Potent Pan FGFR inhibitor	FGFR3 mutations, FGFR2/3 fusions	II

mAb: monoclonal antibody; TKi: Tyrosine Kinase inhibitors.
